# Mutation m.15923A>G in the *MT-TT* gene causes mild myopathy – case report of an adult-onset phenotype

**DOI:** 10.1186/s12883-018-1159-4

**Published:** 2018-09-20

**Authors:** Mikko Kärppä, Laura Kytövuori, Markku Saari, Kari Majamaa

**Affiliations:** 1 Research Unit of Clinical Neuroscience, University of Oulu, P.O. Box 5000, FI-90014 Oulu, Finland; 2 Medical Research Center Oulu, Oulu University Hospital and University of Oulu, Oulu, Finland; 3 Department of Neurology, Oulu University Hospital, P.O. Box 20, FI-90029 OYS Oulu, Finland; 4 Turku Centre for Biotechnology, Cell Imaging Core, University of Turku, FI-20520 Turku, Finland

**Keywords:** Case report, Mitochondrial diseases, Mitochondrial tRNA^Thr^, Neuromuscular disorders, Single-fibre analysis

## Abstract

**Background:**

Only five patients have previously been reported to harbor mutations in the *MT-TT* gene encoding mitochondrial tRNA threonine. The m.15923A > G mutation has been found in three severely affected children. One of these patients died within days after birth and two had a phenotype of myoclonic epilepsy with ragged red fibers (MERRF) in early childhood. We have now found the mutation in an adult patient with mild myopathy.

**Case presentation:**

The patient is a 64-year-old Finnish man, who developed bilateral ptosis, diplopia and exercise intolerance in his fifties. Family history was unremarkable. Muscle histology showed cytochrome c-oxidase (COX) negative and ragged red fibres. The m.15923A > G mutation heteroplasmy was 33% in the skeletal muscle and 2% in buccal epithelial cells. The mutation was undetectable in the blood. Single-fibre analysis was performed and COX-negative fibres had a substantially higher heteroplasmy of 92%, than the normal fibres in which it was 43%.

**Conclusions:**

We report the fourth patient with m. 15923A > G and with a remarkably milder phenotype than the previous three patients. Our findings and recent biochemical studies suggest that the mutation m.15923A > G is a definite disease-causing mutation. Our results also suggest that heteroplasmy of the m.15923A > G mutation correlates with the severity of the phenotype. This study expands the catalog of the phenotypes caused by mutations in mtDNA.

## Background

Mutations in mitochondrial DNA (mtDNA) were found to cause diseases for the first time in 1988 [1, 2], and since then, the number of new mutations and associated phenotypes has continuously increased. MtDNA contains 22 tRNAs, 2 rRNAs and 13 protein-coding genes and disease-causing mutations have been reported in all of them. Mitochondrial tRNA mutations are a well-established cause of mitochondrial disorder, and most of the patients carry the m.3243A > G mutation in *MT-TL1*. The prevalence of mt-tRNA mutations has been estimated to be 4.3 per 100,000 in U.K. [3]. Among these mutations, the prevalence of m.3243A > G solely has been reported to be 3.5 per 100,000. Interestingly, the prevalence is several fold, 16.3 per 100,000, in Finland [4].

While many tRNA genes seem to be mutational hotspots in patients with mitochondrial disorder, the mitochondrially encoded tRNA threonine (*MT-TT*) has been reported to be mutated in five patients only [5–10]. The m.15923A > G has previously been found in three patients; an infant dying from multisystem failure at the age of 2.5 days [5, 6], and two children with myoclonic epilepsy and ragged-red fibers (MERRF) syndrome [8, 10]. We report here the fourth patient with m.15923A > G and the first with an adult-onset phenotype.

## Case presentation

### Patient

The patient was a 64-year-old man, who had bilateral ptosis, diplopia and exercise intolerance. His early development had been normal and currently he had no regular medication. Ocular symptoms had started to develop at the age of 54 years, the right eye had been operated due to squint at the age of 58 years and ptosis surgery had been performed on the right at the age of 63 years. He had right clubfoot, which had been regarded as a complication of vaccination at the age of two years. There was muscle atrophy in the right leg and the leg movements were restricted. His parents, his seven siblings and his son were healthy. Patient’s maternal uncle had ocular symptoms and, interestingly, uncle’s granddaughter had ptosis and a 7.5 kb deletion in mtDNA.

On neurological examination, the patient limped slightly because of the right clubfoot. Ptosis was moderate on the right and mild on the left. Vertical gaze paresis and a slight restriction in horizontal movements was noted in both eyes. Otherwise, muscle examination was normal. Ankle reflexes were absent, while other tendon reflexes were normal.

Routine laboratory values including creatine kinase were normal. Blood lactate was 1.16 mmol/l (reference values 0.33–1.33 mmol/l) and pyruvate was 84 μmol/l (reference values 30–80 μmol/l). Brain MRI showed minimal nonspecific white matter lesions in the frontal lobe. Polyphasic units in frontal and nasal muscles were found in electromyography. Myasthenia gravis was first diagnosed at the age of 60 years and pyridostigmine was initiated. Because the treatment did not alleviate symptoms and all myasthenia studies were negative, treatment was discontinued. Lambert-Eaton myasthenic syndrome was excluded and *PABP2* gene test for repeat expansion causing dominantly inherited oculopharyngeal muscle dystrophy was negative.

**Fig. 1:**
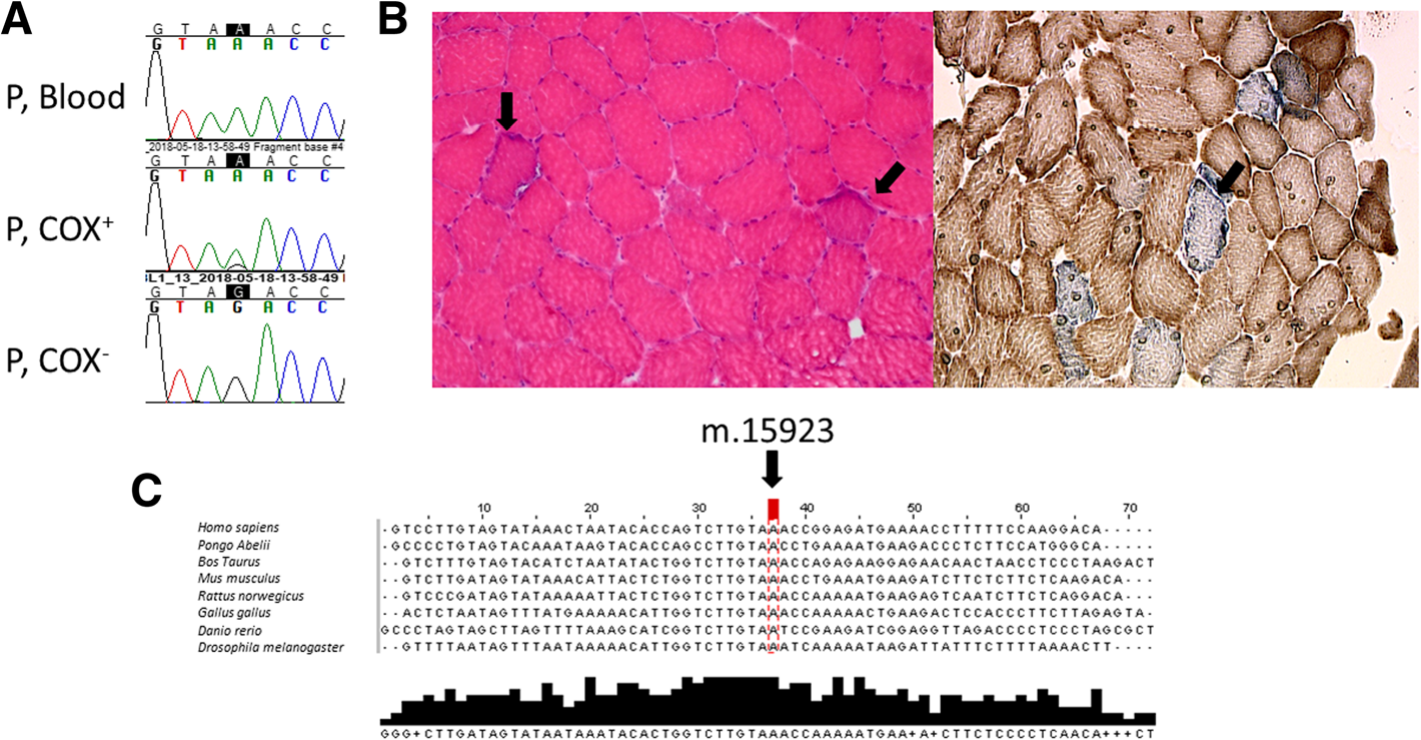
Mutation m.15923A > G is situated in a conservative position and causes typical mitochondria myopathy. **a** Sequence chromatograms showing variable heteroplasmy. P, patient; COX^+^, biochemically normal fibers; COX^−^, cytochrome c-oxidase negative fibers. **b** Histology stainings. Left panel: Hematoxylin & eosin staining showing ragged red fibers. Right panel: Cytochrome c-oxidase staining showing COX-negative (blue) fibers. Arrows denote biochemically abnormal fibers. **c** Clustal Omega [15] alingnment for multiple sequences showing complete conservation between species

Muscle biopsy from *vastus lateralis* was compatible with mitochondrial myopathy (Fig. 1). Ten percent of the muscle fibers were COX-negative and few RRFs were found as well. Ultrastructural examination revealed an increased number of mitochondria and changes in the internal structure of mitochondria.

### Molecular methods and muscle histology

DNA of blood leucocytes and buccal epithelial cells was extracted by using QIAamp DNA Blood Mini Kit (QIAGEN, Hilden, Germany) and that of muscle by using Wizard® Genomic DNA purification kit (Promega Corporation, Madison, WI). Mitochondrial DNA was amplified and sequenced in twelve overlapping fragments. The amplification reactions were done by using *Phire Hot Start II* DNA polymerase (Thermo Fisher Scientific, Waltham, MA, U.S.A.) according to the provided protocol. Sequencing was carried out at Biocenter Oulu sequencing core facility.

The muscle sample for histological staining was fresh-frozen and cryostat sections (5 μm) were stained with routine histochemical techniques [11]. The stainings included hematoxylin and eosin and combined cytochrome c-oxidase and succinate dehydrogenase (COX-SDH). Laser-capture microdissection of COX-SDH stained frozen sections was done using Carl Zeiss P.A.L.M. microscope (Microlaser Technologies GmbH, Bernried, Germany) in Turku Centre for Biotechnology, University of Turku and Åbo Akademi University. Ten COX-negative and ten COX-positive fibers were collected into Carl Zeiss AdhesiveCap tubes (Carl Zeiss Gmbh, Göttingen, Germany) and DNA was released incubating fibers 30 min in 65 °C in lysis buffer containing 200 mM potassium hydroxide and 50 mM dithiothreitol followed by neutralization step with 900 mM Tris-HCl, pH 8.3. Amplification was carried out using Phusion High-Fidelity DNA polymerase (Thermo Fisher Scientific). Heteroplasmy was determined by cloning using CloneJET PCR Cloning Kit with blunt-end cloning protocol and DH5α competent cells (Thermo Fisher Scientific). Colony screening was done by using FastDigest *XmiI* (Thermo Fisher Scientific). XL-PCR for whole mtDNA amplification was carried out using Phusion High-Fidelity DNA polymerase with GC Buffer according to the original protocol (Thermo Fisher Scientific).

We found the m.15923A > G mutation in *MT-TT* in the skeletal muscle of the patient. The heteroplasmy was determined and, interestingly, the mutation was undetectable in the blood of the patient, while it was present with a 33% heteroplasmy in the skeletal muscle and with 2% heteroplasmy in the buccal mucosa. In pooled COX-negative fibers, the heteroplasmy was 92% while it was 43% in biochemically normal fibers. The investigation of mtDNA deletions remained negative.

## Discussion and conclusions

We found the m.15923A > G mutation in an adult patient with ptosis and exercise intolerance. The pathogenicity of the mutation has been unclear, because the mutation has been found only in three patients before our study. Recent studies have shown, however, that m.15923A > G affects post-translational modification of tRNA threonine [10, 12]. The nucleotide in position 38 (m.15923A) has been shown to be crucial in N^6^-threonylcarbamoyladenosine (t^6^A) modification that occurs in position 37, and decreased level of t^6^A-modified tRNA has been detected in patient cell lines [10]. In addition, according to the Yarham scoring [13], the m.15923A > G is classified as definitely pathogenic. The score includes evaluation of nucleotide site conservation, heteroplasmy and mutation segregation with disease, and evidence of biochemical defect from single-fiber studies and mitochondrial complex activity measurements [13].

Three children harboring m.15923A > G have been reported to suffer from severe mitochondrial disorder (Table 1) [5, 6, 10]. Patient 1 was a girl who died 2.5 after birth of a fatal cardiopulmonary arrest [5, 6]. The mother had a history of five miscarriages and a delivery of a boy who died within two days. The two pregnancies and deliveries and the first 24 h of the infants had been normal. The mutation was heteroplasmic in the girl, but samples were not available from her deceased brother. Patient 2 was a girl with symptoms from early childhood [8]. The patient had exercise intolerance, vomiting and generalized seizures during her early childhood. She developed deafness, migraine, retinitis pigmentosa, and cognitive delay. Her symptoms were progressive. Mutation heteroplasmy was 78% in the muscle, 10% in the blood and 18% in buccal mucosa. Patient 3 was a 15-year-old girl with bilateral hearing impairment as the first manifestation at the age of 6 years [10]. At the time of the latest clinical examination, she had retinitis pigmentosa, lactic acidosis, myoclonic epilepsy, proteinuria and migraine suggesting a MERRF syndrome.

**Table 1:** Patients harboring the m.15923A > G mutation in the *MT-TT* gene

	Patient 1	Patient 2	Patient 3	Patient 4
Reference	[5, 6]	[8]	[10]	present study
Age at onset	Day 2	Childhood	6 years	54 years
Age at the latest examination	Died at the age of 56 h	19 years	15 years	64 years
Hypoglycemia	yes	–	–	–
Lactic acidosis	yes	yes	yes	–
Multisystem failure	yes	–	–	–
Short stature	–	–	yes	–
Hearing impairment	–	yes	yes	Age-related
Exercise intolerance	–	yes	–	yes
Muscle weakness	–	yes	–	yes
Generalized seizures	–	yes	yes	–
Myoclonic seizures	–	yes	yes	–
Cognitive delay	–	yes	–	–
Migraine	–	yes	yes	–
Ataxia	–	yes	–	–
Dysmetry	–	yes	–	–
Dysarthria	–	yes	–	–
Vomiting	–	yes	–	–
Retinitis pigmentosa	–	yes	yes	–
Ptosis	–	–	–	yes
Diplopia	–	–	–	yes
MRI	–	Cortical and cerebellar atrophy	Basal ganglia calcifications	Nonspecific white matter lesions in frontal lobe
Family history	Several miscarriages of the mother	Deafness, maternal aunt	Negative	Nonspesific
RRF	n.a.	yes	yes	yes
COX-negative	n.a.	yes	yes	yes
Other	n.a.	Mitochondrial aggregates with abnormal structure	n.a.	Mitochondrial aggregates with abnormal structure
Functional studies	Decreased activity of complexes III and IV	Complex IV normal	Defect in tRNA modification	COX-negative fibres had higher mutation heteroplasmy than normal fibres
Heteroplasmy				
Blood	n.a.	10%	n.a.	< 1%^a^
Skeletal muscle	n.a.	78%	> 95%^b^	33%
Buccal epithelium	n.a.	n.a.	n.a.	2%

*RRF* Ragged red fibres, *COX* Cytochrome c-oxidase, *n.a*., Not analyzed

^a^Undetectable by cloning, 100 colonies

^b^The mutation was reported to be homoplasmic in Sanger sequencing

Decreased activity of OXPHOS complexes is a common finding in patients with mitochondrial disorder. The activity of complexes III and IV have been reported to be decreased in the muscle, liver and kidney of patient 1 [5, 6], whereas the activity of complex IV was normal in the muscle of patient 2 with mutation heteroplasmy of 78% [8]. Unfortunately, lack of remaining muscle prevented us from carrying out these analyses. However, previous studies and our single-fibre analysis suggest that the heteroplasmy required to cause an OXPHOS defect is very high and, therefore, the heteroplasmy of 33% in our patient may not be sufficient to cause the defect.

Pathogenic mutations in *MT-TT* are rare. In addition to m.15923A > G in the three patients, two other mutations have been found previously in single patients [5–10]. The m.15915A > G mutation has been found in a 16-year-old boy, whose symptoms started at the age of 8 years [7]. He had muscle weakness, hearing impairment, seizures, ptosis, intellectual disability, growth failure, and mitochondrial myopathy in muscle histology. The m.15933G > A mutation has been found in an adult patient, who had exercise-induced muscle weakness, myalgia, dysphagia and ptosis with mitochondrial myopathy in the skeletal muscle [9].

We conclude that the pathogenicity of the m.15923A > G mutation is now confirmed on the basis of the following facts: The mutation has been found in three severely affected children [5, 6, 8] and we describe the first adult patient harboring the mutation and with a mild mitochondrial disorder. The mutation has not been reported in population controls or listed among polymorphisms in MITOMAP database [14]. Mutation heteroplasmy has been shown to be high in the children with fatal mitochondrial disorder, while it was lower in our patient with a milder phenotype. COX-negative and ragged red fibers have been found in the skeletal muscle in all the four patients and decreased OXPHOS enzyme activities have been reported in the most severely affected infant. Differences in mutation load between biochemically deficient and normal fibers in our study further confirm the functional consequences of the mutation.

## Abbreviations

COX: Cytochrome c-oxidase
mtDNA: Mitochondrial DNA
*MT-TT*: Mitochondrialally encoded transferRNA threonine
OXPHOS: Oxidative phosphorylation
